# Value of free/total prostate-specific antigen (f/t PSA) ratios for prostate cancer detection in patients with total serum prostate-specific antigen between 4 and 10 ng/mL: A meta-analysis

**DOI:** 10.1097/MD.0000000000010249

**Published:** 2018-03-30

**Authors:** Yan Huang, Zhen-Zhen Li, Ya-Liang Huang, Hong-Jun Song, You-Juan Wang

**Affiliations:** a Health Management Center, West China Hospital of Sichuan University; b Department of Nephrology and Rheumatology, Affiliated Hospital/Clinical Medical College of Chengdu University; c Out-patient Department, West China Hospital of Sichuan University, Chengdu, Sichuan, China.

**Keywords:** diagnosis, f/t PSA, prostate carcinoma, PSA, sensitive, specificity

## Abstract

**Background:**

Prostate carcinoma is a common disease that occurs in men over 50 years old. Many studies have explored the effect of free/total prostate-specific antigen (f/t PSA) ratio in monitoring prostate cancer. We conducted a meta-analysis to identify the accuracy of the f/t PSA ratio in the diagnosis of prostate cancer in patients who have PSA levels of 4 to 10 ng/mL.

**Methods:**

Databases searched included PubMed and OVID databases, from inception to March 2017, after a systematical review, sensitivity, specificity, and other measures of accuracy of the f/t PSA ratio in the diagnosis of prostate cancer were pooled. We used summary receiver operating characteristic curves to summarize overall test performance.

**Results:**

Fifteen case–control studies from 14 articles were identified. The results indicated that the sensitivity of the f/t PSA ratio in the diagnosis of prostate cancer ranged from 0.5 to 0.94 (pooled sensitivity 0.70, 95% CI: 0.67–0.72), whereas its specificity ranged from 0.31 to 0.93 (pooled specificity 0.55, 95% CI: 0.57–0.60). The positive likelihood ratio was 1.85 (95% CI: 1.56–2.20), negative likelihood ratio was 0.42 (95% CI: 0.34–0.53), and diagnostic odds ratio was 4.81 (9.53% CI: 3.33–6.94).

**Conclusions:**

The f/t PSA ratio determination has a low sensitivity and specificity for the diagnosis of prostate cancer; it would not be useful for the diagnosis of prostate cancer by itself. The results of f/t PSA ratio measurements should refer to the clinical manifestations and the results of conventional tests such as biopsies.

## 1 Introduction

Prostate carcinoma is a common disease that occurs in men over 50 years old; serum prostate-specific antigen (PSA), which is produced by all types of prostate tissue, is one of the most important biomarkers for detecting prostate cancer, guiding decisions about biopsies of the prostate and offering a way to monitor disease progression.^[1,2]^ Total PSA (tPSA) includes unbound and bound (or complexed) PSA forms; it refers to the sum of all immunologically detectable forms of serum PSA. Because free PSA (fPSA) became a biomarker identification in 1991,^[3]^ many studies have shown that PSA has a high sensitivity but a low specificity, which can result in unnecessary biopsies, especially in patients with benign disease, and cancers overlap when the tPSA is moderately elevated. Because the free/total (f/t PSA) ratio appears to be most clinically useful when PSA reaches levels of 4 to 10 ng/mL, detecting the free/total (f/t PSA) ratio can improve the specificity in monitoring prostate cancer and decrease the number of negative biopsies in patients.

Some studies have reported that the f/t PSA ratio provides high diagnostic sensitivity (94%) and specificity (93%). Other studies, however, have reported much lower corresponding values of 75% and 32%. Because the results are inconclusive, we meta-analyzed the available literature to gain a comprehensive status of the diagnostic usefulness of the f/t PSA ratio in prostate carcinoma when patients had total serum prostate-specific antigen between 4 and 10 ng/mL. To the authors’ knowledge, this is the first meta-analysis to investigate the diagnostic usefulness of the f/t PSA ratio in prostate carcinoma.

## 2 Methods

In accordance with the guidelines of the Preferred Reporting Items for Systematic Reviews and Meta-Analyses statement,^[4]^ a prospective protocol of objectives, eligibility criteria, literature search strategies, and methods of statistical analysis was prepared.

### 2.1 Literature search strategy

According to the study protocol, PubMed and OVID databases were searched for meta-analyses existed that related to value of free/total prostate specific antigen ratios for prostate cancer detection, no article was found. A comprehensive electronic database search was performed to identify articles published up to May 2017. In PubMed, the search string was ((((*free prostate specific antigen* OR *total prostate specific antigen* OR *prostate cancer*) AND *sensitivity*) AND *specificity*) AND *diagnosis*). In OVID, the search string was: “*free prostate specific antigen*” OR “*total prostate specific antigen*” OR “*free/total prostate specific antigen*” AND “*prostate cancer*” AND “*sensitivity*” AND “*specificity*” AND “*diagnosis*”. Only English-language articles were included. We evaluated potentially relevant articles in references by examining their titles and abstracts manually, and all the studies matching the eligibility criteria were included. All analyses were based on previously published studies; thus, no ethical approval and patient consent are required.

### 2.2 Inclusion criteria

For a study to be included in our meta-analysis, it might meet all the following criteria: Information about the sensitivity and specificity of f/t PSA for the diagnosis of prostate cancer and number of patients was complete. Case–control design was performed. Diagnostic criteria were clear. All the PSA levels in the study were 4 to 10 ng/mL. Unpublished data, insufficient data, case reports, letters to editor, abstracts, and review articles were excluded.

### 2.3 Data extraction

According to the prespecified protocol, all data were extracted by 2 authors independently. The following data were extracted from each eligible study by using a standardized data collection form: the first author's name; the country where the study was conducted; the year of publication; the level of PSA; the cutoff values of f/t PSA; the sensitivity and specificity of f/t PSA for the diagnosis of prostate cancer; and the true positive, false positive true negative, and false negative in each study.

### 2.4 Methodological quality assessment

The methodological quality of studies was assessed using the QUADAS-2 checklist.^[5]^ QUADAS-2 assesses risk of bias in 4 parts: patient selection, reference standard, index test, flow, and timing. It assesses applicability concerns in 3 parts: patient selection, index test, and reference standard. Two authors read each study and scored them independently; disagreement between the 2 authors was settled by discussion with the third author and resolved by consensus.

### 2.5 Statistical analysis

Standard methods recommended for meta-analyses of diagnostic test evaluations^[6]^ were used. STATA version 12.0 and Meta-DiSc (XI Cochrane Colloquium, Barcelona, Spain) software were used for the statistical analyses.^[7]^ The following measures of test accuracy were computed for each included study: sensitivity, specificity, positive likelihood ratio (PLR), negative likelihood ratio (NLR), and diagnostic odds ratio (DOR). Overall diagnostic performance was assessed from summary receiver operating characteristic (SROC) curves.^[8]^ These curves were pooled for the studies using sensitivity and specificity based on the single-test threshold identified within the same study.^[9]^ The random-effect model was used for the meta-analysis.^[10,11]^ To assess statistically significant variability (heterogeneity) across studies, we used Chi-squared and Fisher's exact tests. The *I*^*2*^ exceeding 50% is considered to indicate the presence of heterogeneity. If significant heterogeneity existed among studies, meta-regression analysis was performed using covariates reported in most included studies: cutoff values, ethnicity (Asian vs Caucasian), study design (prospective vs. retrospective), publication year (before 2007 vs after 2007). Publication bias was assessed through the visual inspection of funnel plots and with tests of Begg rank correlation.^[12]^*P* < .05 was considered representative of a significant statistical publication bias.

## 3 Results

Initially, a total of 202 potentially eligible studies were identified. After the screening of titles and abstracts, 184 articles were excluded. The remaining 18 articles were read in full and then 4 articles were removed because they did not show the sufficient data of cutoff values. In the 14 eligible articles, one study^[17]^ was related to 2 case–control groups and each of them reported sufficient data, so we treated the 2 groups as 2 independent studies.^[13–26]^ Therefore, the current meta-analysis included 15 studies from 14 publications,^[13–26]^ The clinical characteristics of each study are shown in Table 1.

**Table 1:**
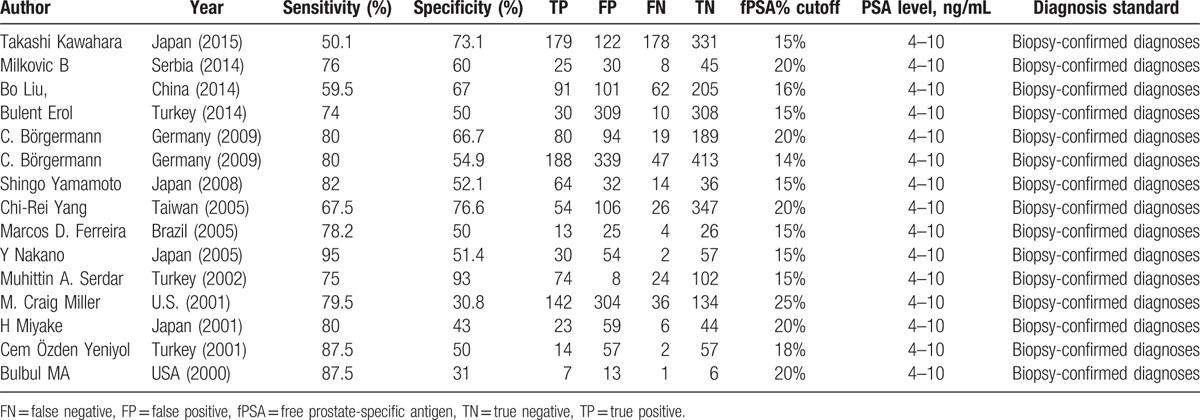
Characteristics of studies included in the meta-analysis.

### 3.1 Study characteristics

The total sample size in the 15 studies was 5406, comprising 1453 patients with prostate cancer and 3953 without it. Prostate cancer was diagnosed by biopsy. The cutoff values of f/t PSA are varied. The QUADUS-2 score in each study was relatively high, means each of them has a low risk of bias (Table 2).

**Table 2:**
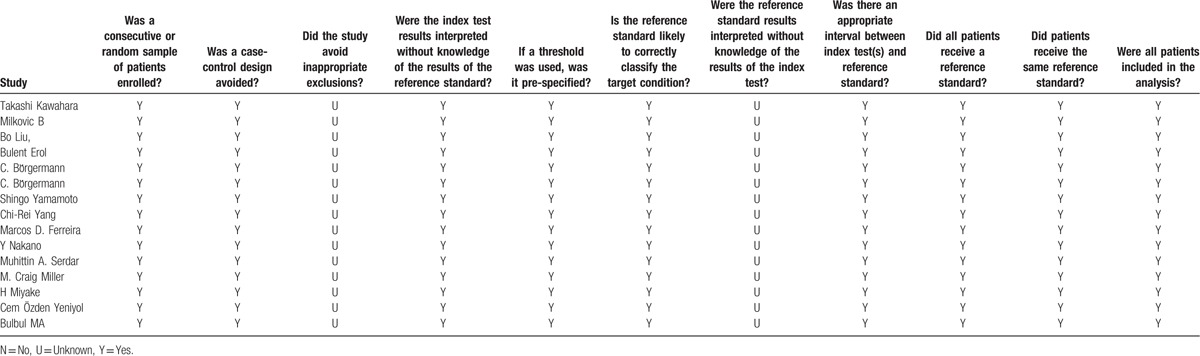
QUADUS-2 score of each included article.

### 3.2 Diagnostic accuracy

**Figure 1:**
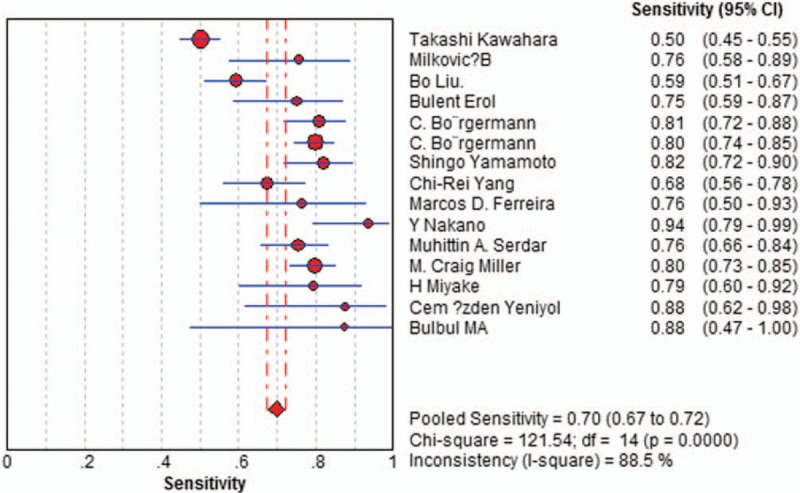
Forest plot of estimates of sensitivity for f/t PSA in the diagnosis of prostate cancer. Point estimates of sensitivity from each study are shown as solid circles, the size of which reflects the total number of cases and controls. Error bars show 95% confidence intervals. Numbers indicate the reference numbers of the studies. f/t PSA = free/total prostate-specific antigen.

**Figure 2:**
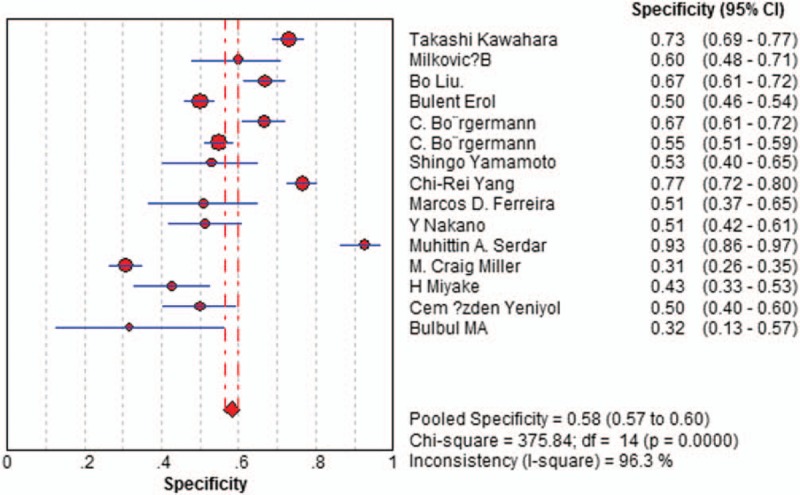
Forest plot of estimates of specificity for f/t PSA in the diagnosis of prostate cancer. Point estimates of specificity from each study are shown as solid circles, the size of which reflects the total number of cases and controls. Error bars show 95% confidence intervals. Numbers indicate the reference numbers of the studies. f/t PSA = free/total prostate-specific antigen.

**Figure 3:**
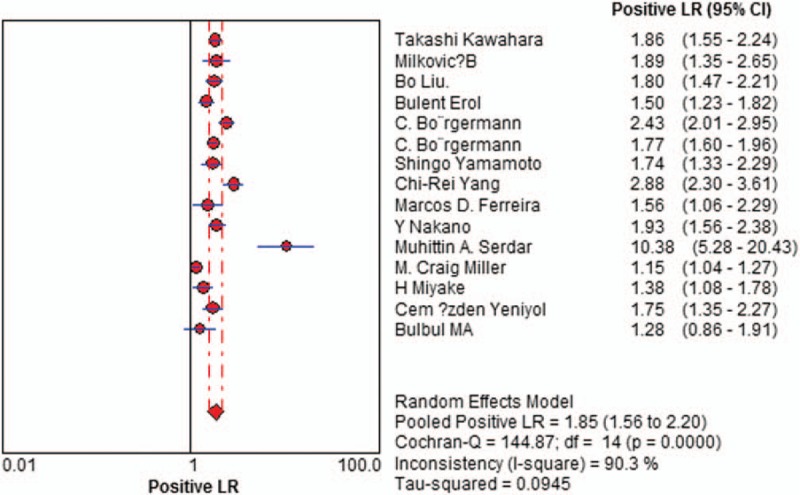
Forest plot of estimates of positive likelihood ratios for f/t PSA in the diagnosis of prostate cancer. Point estimates of positive likelihood ratios from each study are shown as solid circles, the size of which reflects the total number of cases and controls. Error bars show 95% confidence intervals. Numbers indicate the reference numbers of studies. f/t PSA = free/total prostate-specific antigen.

**Figure 4:**
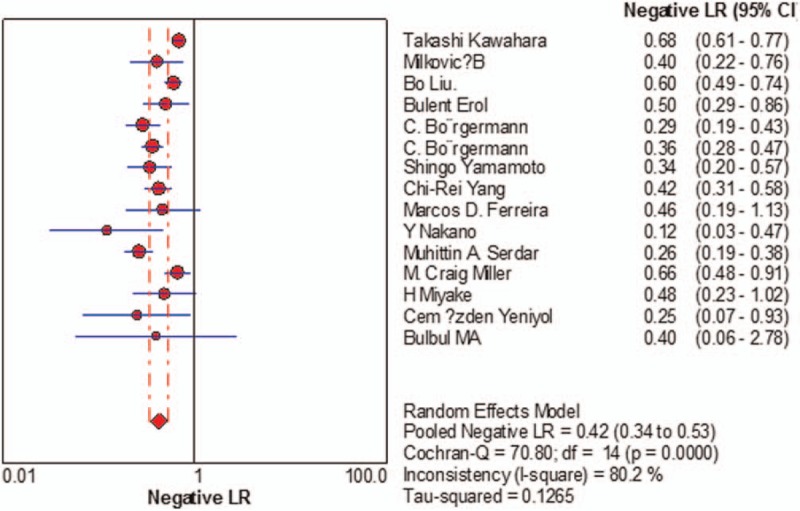
Forest plot of estimates of negative likelihood ratios for f/t PSA in the diagnosis of prostate cancer. Point estimates of negative likelihood ratios from each study are shown as solid circles, the size of which reflects the total number of cases and controls. Error bars show 95% confidence intervals. Numbers indicate the reference numbers of studies. f/t PSA = free/total prostate-specific antigen.

**Figure 5:**
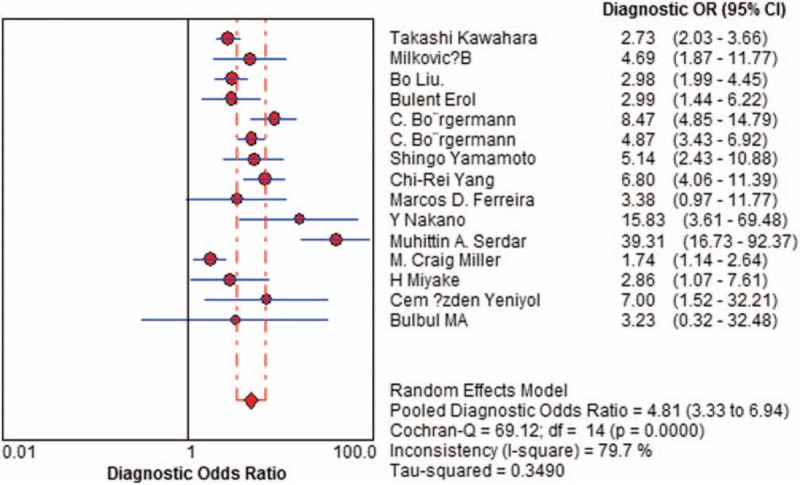
Forest plot of estimates of diagnostic odds ratios for f/t PSA in the diagnosis of prostate cancer. Point estimates of diagnostic odds ratios from each study are shown as solid circles, the size of which reflects the total number of cases and controls. Error bars show 95% confidence intervals. Numbers indicate the reference numbers of studies. f/t PSA = free/total prostate-specific antigen.

Sensitivity of f/t PSA ratio ranged from 0.68 to 0.94 and the pooled value was 0.70 (95% CI: 0.67 to 0.72) (Fig. 1). Specificity ranged from 0.31 to 0.93 and the pooled value was 0.58 (95% CI: 0.57 to 0.60) (Fig. 2). The following summary parameters have also been calculated: PLR was 1.85 (95% CI: 1.56 to 2.20) (Fig. 3), and NLR was 0.42 (95% CI 0.34 to 0.53) (Fig. 4). DOR of f/t PSA ratio for prostate cancer detection was 4.81 (95% CI: 3.33 to 6.94) (Fig. 5). Random-effects model was used in these analyses. I2 was 88.5% for sensitivity, 96.3% for specificity, 90.3% for PLR, 80.2% for NLR, and 79.7% for DOR.

**Figure 6:**
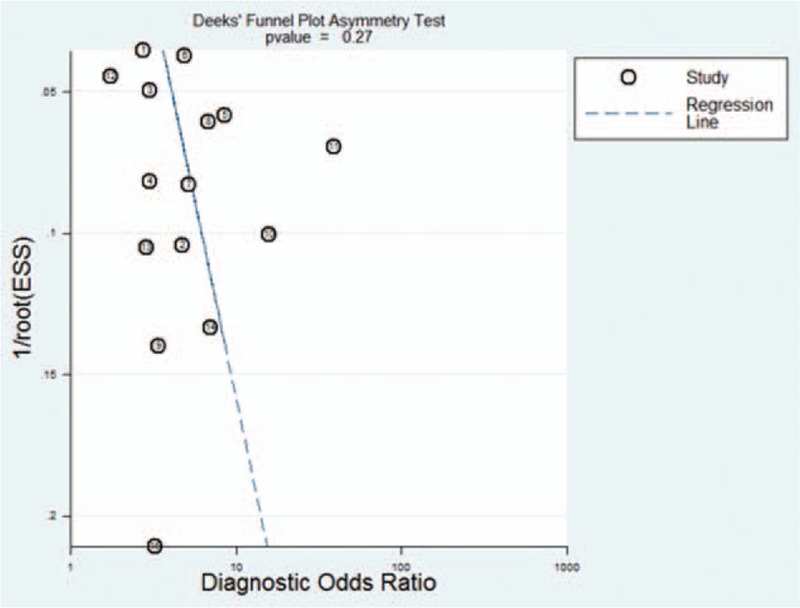
Summary receiver operating characteristic curves for f/t PSA. Each study is depicted as a solid circle, the size of which reflects the total number of cases and controls. f/t PSA = free/total prostate-specific antigen.

SROC curves plots sensitivity against (1-specificity) in individual studies (Fig. 6). The SROC curves positioned not near the desired upper left corner of the plot, and the maximum joint sensitivity and specificity was 0.70, suggesting not a good performance, the area under the curve (AUC) was 0.7617 (SEM 0.0248).

### 3.3 Meta-regression analysis

*I*^2^ for pooled diagnostic performance parameters were high, which indicates significant heterogeneity among the studies of this research. To identify possible reasons for this heterogeneity, we conducted meta-regression to assess the effect of study quality on the relative DOR (RDOR) of the f/t PSA ratio for prostate cancer. The characteristics of the covariates are shown in Table 1. Diagnostic accuracy was not significantly affected by the cutoff value (*P = *.5392), ethnicity (*P = *.4186), study design (*P = *.2514), or publication year (*P = *.2893). The meta-regression results are shown in detail in Table 3.

**Table 3:**
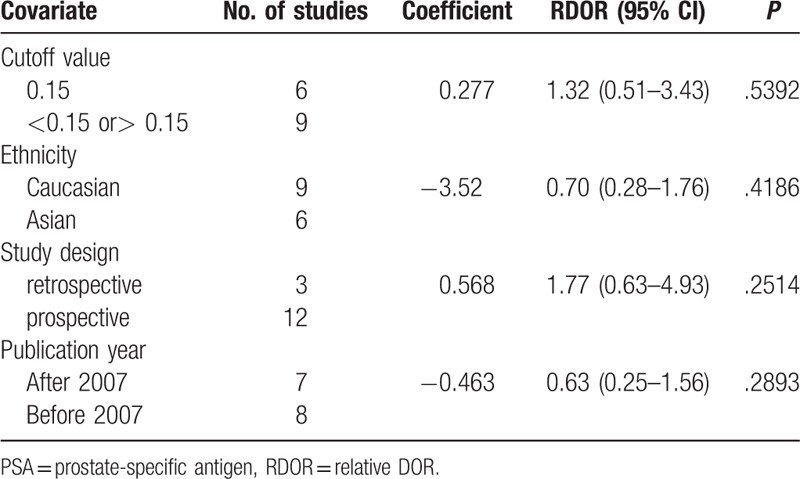
Meta-regression of the diagnostic accuracy of f/t PSA.

### 3.4 Publication bias

**Figure 7:**
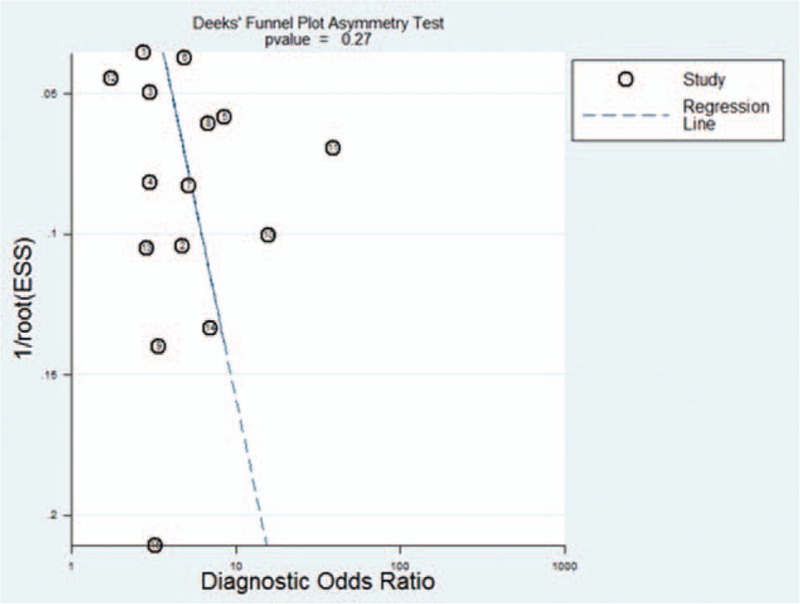
Funnel plot for evaluating publication bias among the twelve studies included in the meta-analysis. The log of the diagnostic odds ratio (DOR) is plotted against the standard error of log DOR; the latter serves as an indicator of sample size. Each article is shown as a solid circle, and the regression line is shown. DOR = diagnostic odds ratio.

Funnel plots showed some asymmetry (Fig. 7); nevertheless, Deeks’ test gave a *P* value of .27, suggesting that our analysis did not have significant risk of publication bias.

## 4 Discussion

Total PSA refers to the sum of all immunologically detectable forms of PSA in serum, including the free PSA (fPSA) and plus-bound PSA forms. For suspicious prostate cancer, transrectal ultrasonography-guided prostate biopsy can provide the gold standard—histological information—but it is invasive and skill dependent. To reduce unnecessary prostate biopsies in benign prostatic hyperplasia patients, some biomarkers such as PSA and fPSA can be used in guiding biopsies, but the lack of sensitivity and specificity restrict their use for detecting prostate cancer alone. As some studies indicated that the f/t PSA ratio could improve sensitivity and specificity in detecting prostate cancer, many researchers paid attention to the value of f/t PSA ratios for prostate cancer detection in recent years. Denham^[27]^ pointed out that the value of f/t PSA ratios had no superiority in discriminating prostate cancer and benign prostatic hyperplasia. However, many scholars, such as Mungan,^[28]^ reported that the ratio of f/t PSA had a benefit in discriminating between prostate cancer and benign prostatic hyperplasia. Our findings are in agreement with Denham.

Diagnostic studies have indicated highly variable sensitivity and specificity when using the f/t PSA ratio, urging us to perform what we deem to be the first meta-analysis to assess the available evidence of the diagnostic usefulness of the f/t PSA ratio in prostate cancer. Our analysis suggests that f/t PSA measurements by themselves are not sufficiently sensitive (0.70) or specific (0.58) and have insufficient accuracy (DOR is 4.81) to diagnose prostate cancer. DOR is the ratio of the odds of positive test results in people with disease relative to the odds of positive test results in people without disease,^[29]^ which combines sensitivity data and specificity data that serve as an aggregate indicator of test accuracy.^[30]^ The SROC curve and the area underneath it show tradeoff between sensitivity and specificity^[29]^ The area under the SROC curve, 0.7617, indicates relatively low-accuracy DOR and SROC curve analysis. Likelihood ratios are more meaningful for measuring diagnostic accuracy in clinical practice.^[31,32]^ Therefore, we meta-analyzed the pooled PLR and NLR. The PLR value of 1.85 suggests that prostate cancer patients have about a twofold higher chance of having f/t PSA above cutoff values than people without prostate cancer; this is insufficient to serve as the sole basis for diagnosing prostate cancer. At the same time, the NLR was 0.42, which means it has a 42% probability that the patient has prostate cancer if the f/t PSA ratio is below cutoff values. This also shows that such a measurement is inadequate for ruling out prostate cancer on its own.

Heterogeneity among the included studies determines the reliability of meta-analyses; we found significant heterogeneity among the studies of our meta-analysis; therefore, the results should be interpreted with caution. We checked the 14 articles more carefully to find the possible reasons for this heterogeneity and found that in each included study, the prostate cancer was diagnosed based on histology, and the QUADUS-2 score in each study was relatively high, but the cutoff value, ethnicity, study design, and publication year were varied. However, the meta-regression results reveal that the diagnostic accuracy was not significantly affected by these factors. Therefore, the basis for the heterogeneity in our meta-analysis is unclear. The studies included in our meta-analysis varied in the age of the subjects; the stages of prostate cancer in each patient of the studies were different as prostate cancer advances from stage T1 to T4. Benign prostatic hyperplasia in the control groups of the studies were also at the same stage. These factors may affect diagnostic accuracy. In any case, further large studies are needed to verify our findings.

In our meta-analysis, the cutoff values for f/tPSA ratio of the included studies varied and were usually between 0.14 and 0.25. As there are no international standards of cutoff value right now, most studies included in our meta-analysis did not describe the immunometric assay, which is used for the detection of serum PSA and fPSA levels, and its analytical sensitivity. According to different country, ethnicity, age, detection equipment, disease severity and complication, the cutoff values for the f/t PSA ratio are likely to vary with different clinical context. However, our meta-regression suggested that different cutoff values did not significantly affect the diagnostic accuracy of the f/t PSA ratio. Nevertheless, researchers should be aware of the truth that it is better to determine different cutoffs for different types of patients or different clinical contexts. In the further work, we should aim at identifying the optimal cutoff values that provide the highest diagnostic accuracy, as well as determining different cutoffs for different types of patients with various clinical contexts.

There are some limitations in our study. Firstly, we excluded publications without sufficient data, such as conference abstracts, reviews, and case reports. Secondly, we only included English-language articles, which may bias our results. Thirdly, we could not search for unpublished articles or articles that were not indexed in our set of databases. However, no significant risk of publication bias was detected in our our funnel plots.

## 5 Conclusion

F/t PSA ratio determination has a low sensitivity and specificity for the diagnosis of prostate cancer; it would not be specific or sensitive enough to use on its own. The results of the f/t PSA ratio must always be combined with other established diagnostic methods.

## Author contributions

**Conceptualization:** Y. Wang, Y. Huang.

**Formal analysis:** Y. Huang, Y. Wang.

**Investigation:** Yan Huang, Yangliang Huang.

**Methodology:** Yan Huang, Yangliang Huang.

**Project administration:** Y. Wang.

**Resources:** H. Song, Y. Huang, Z. Li.

**Software:** H. Song, Y. Huang, Z. Li.

**Supervision:** Y. Wang.

**Validation:** Z. Li, Y. Wang.

**Writing – original draft:** Y. Huang.

**Writing – review & editing:** Y. Wang.
